# Ectopic primary type A thymoma located in two thoracic vertebras: a case report

**DOI:** 10.1186/1471-2407-10-322

**Published:** 2010-06-23

**Authors:** Ferdinando Marandino, Carmine Zoccali, Nicola Salducca, Mirella Marino, Paolo Visca

**Affiliations:** 1 Oncological Orthopaedics Department, Muscular-skeletal Tissue Bank, Regina Elena Cancer Institute, Via Elio Chianesi 53, 00144, Rome, Italy; 2 Department of Surgical Pathology, Regina Elena Cancer Institute, Via Elio Chianesi 53, 00144, Rome, Italy

## Abstract

**Background:**

The thymus arises in the ventral portion of the third and fourth pharyngeal pouch. It descends into the anterior mediastinum at 6^th ^week of gestation. Any errors occurring during this process can cause dissemination of aberrant nodules that are responsible for most atypical thymomas.

**Case Presentation:**

The authors report a unusual case of type-A thymoma located in D10 and D11 vertebral bodies.

The histology showed a uniform growth of short, spindle shaped, mitotically inactive cells. A few small, normal lymphocytes were seen scattered or in small groups. The immunohistochemical investigation for neuroectodermal, neuroendocrine, vascular and muscular markers were negative. It also confirmed the presence of CD3+, CD5+ T lymphocytes and the absence of immature T-lymphocyte markers.

**Conclusions:**

The case described shows a thymic hystogenesis for spindle cell tumours. To our knowledge no other cases of vertebral thymomas have been described in international literature.

## Backgroud

The thymus arises in the ventral portion of the third and fourth pharyngeal pouch. It descends into the anterior mediastinum at 6^th ^week of gestation. Any errors occurring during this phase can cause dissemination of aberrant nodules that are responsible for most uncommon thymomas [1]. Seldomly do thymomas appear in other locations, such as the base of the skull [2], pulmonary parenchyma [3], pleura [4], and in the middle mediastinum [5]. We describe here the first case of intra-vertebral type-A thymoma where a thorough immunohistochemical assessment has been carried out.

### Case Presentation

A 59-year-old woman presenting with a tumour mass located in D11 with partial invasion of D10 causing middle back pain one year before.

There was no family history of bone lesions or bone tumours. There were no general symptoms, such as an increase in temperature or loss of weight or appetite.

**Figure 1:**
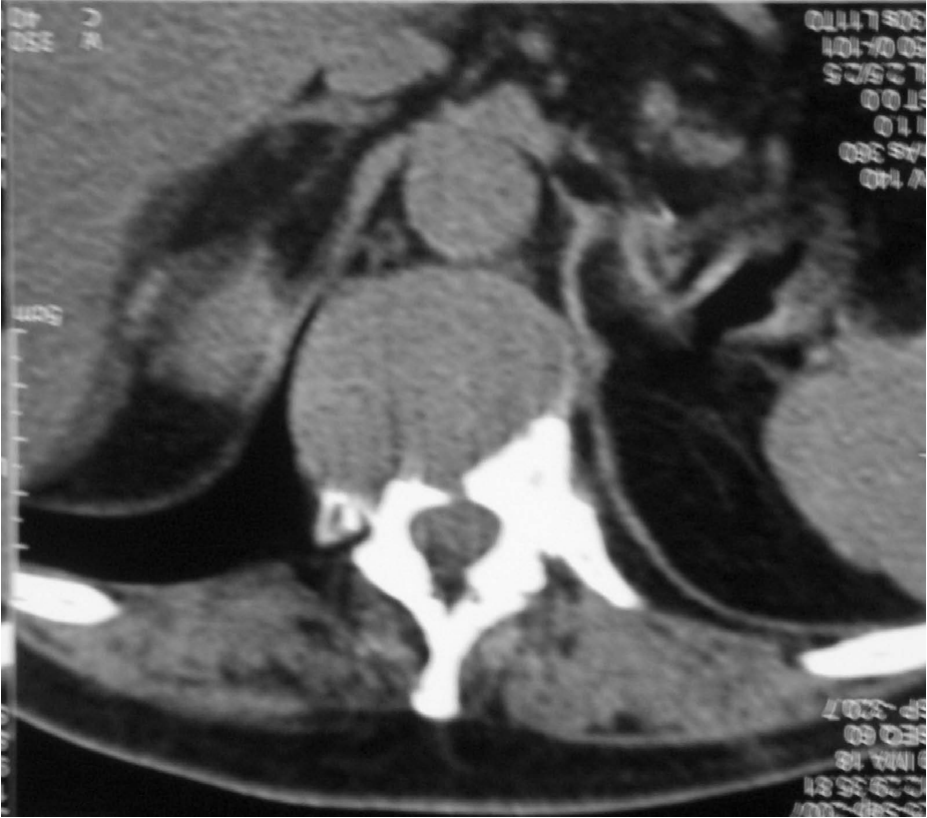
**Chest CT of lesion**. Chest CT showing osteolytic lesion of D11 eroding anterior cortex and superior endplate and involving superior vertebral body.

**Figure 2:**
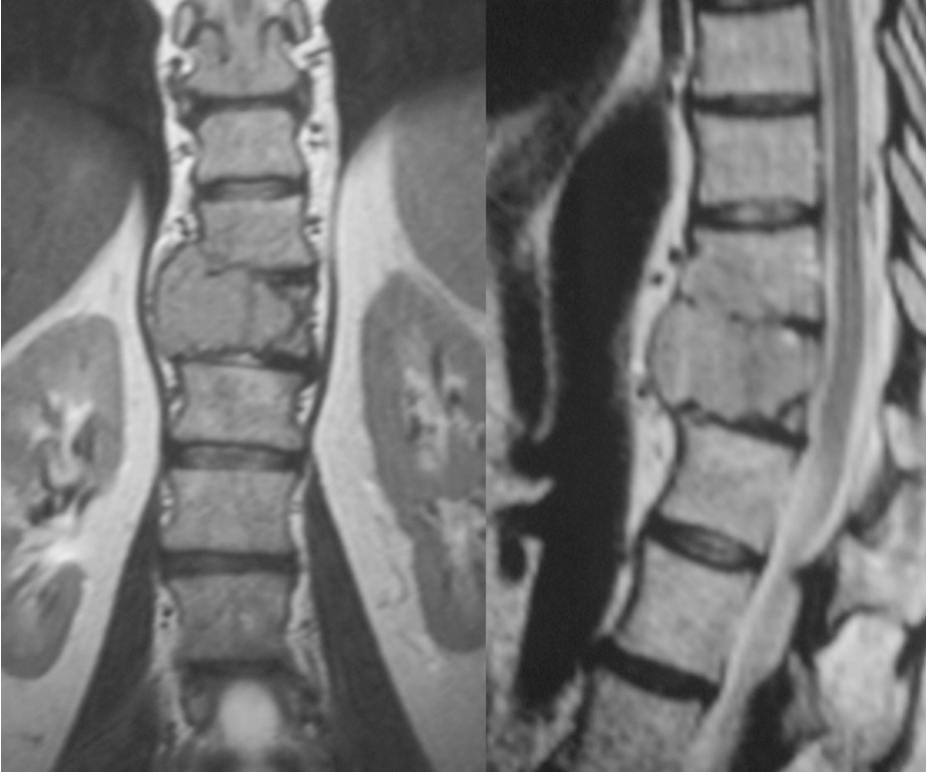
**MRI of lesion**. T2-weighted MRI image showing the tumour in D11 involving the upper vertebra as well.

Clinical examination results showed localized pain increasing with compression. A systemic examination was performed and tested negative. Laboratory studies were not helpful. X-rays revealed an osteolytic lesion eroding the anterior cortex, the superior endplate involving the upper vertebra (fig. 1). The CT-scan and MRI (fig. 2) confirmed the subtotal substitution of the vertebral body. A CT-scan performed about one year before confirmed similar results. The bone scan indicated a slight increase in local uptake and no sign of bone metastasis, the condition was confirmed by a total-body CT-SCAN. While X-rays and CT-scans of the chest were normal.

The mass was angiographically quite avascular suggesting a low grade lesion, according to the FDG-PET which demonstrated low metabolic activity and no signs of dissemination. As a tumour was suspected a CT-guided needle-biopsy was performed under local anesthesia through the left pedicle.

Histopathological tests showed a mitotically inactive spindle cell tumour, however, no definite diagnosis could be performed as material was scant and the immunohistochemical findings were inconsistent.

Hemangiopericytoma and paraganglioma were excluded because of low vascular grade; a metastasis from renal carcinoma, before hypothesized was excluded because contrasted with angiography and FDG-PER-FDG pattern. Having an uncertain diagnosis, a tumour mass has to be treated as a primitive tumour with therapeutic intent thus an en-block vertebral resection was proposed. The patient was informed about the high surgical and post-surgical risks and would not accept undergoing the procedure, so therefore a less dangerous palliative surgical procedure was chosen. During the first surgical operation a laminectomy and a posterior stabilization were performed without carrying out a biopsy, so that the tumour would not spread to the posterior access.

Curettage and stabilization with a titanium cage, cement and anterior plate was performed about two months later by intercostals-lateral approach.

Postoperative inpatient time followed without any problems and the patient was discharged after ten days in order to start rehabilitation.

Histopathological features were done on the curetted material, and pin-pointed the localization of ectopic thymomas so that a meticulous immunohistochemical test panel was applied to confirm the hypothesis.

An indirect immunoperoxidase staining was performed on 2 μm formalin fixed paraffin embedded sections by a streptavidin-biotin enhanced immunoperoxidase technique (Super Sensitive MultiLink, Novocastra, Menarini Florence, Italy) in an automated auto-stainer (Bond Max , Menarini) using monoclonal and polyclonal antibodies directed to the following antigens: CD99 (clone MIC2, UCS Diagn. Rome, Italy), chromogranin A (clone DAK-A3, Dako, Milan, Italy), Smooth Muscle Actin (clone 1A4, Dako) without any retrieval pre-treatments, HMB45 (clone HMB45 Dako), Synaptophysin (clone SY38, Dako), CD31 (clone JC/70A, Dako), CD34 (clone QBEND, Novocastra, Menarini, Florence, Italy), pan-cytokeratin (clone AE1/AE3, Novocastra), keratin 19 (clone RCK108, Dako), keratin 7 (clone OVTL12/30, Dako), Vimentin (clone V9, Dako), CD56 (clone BC56C04, UCS), EMA (clone E-29, Dako), Bcl2 (clone 124, Dako), CD20 (clone L26, Menarini), CD1a (clone 010, Dako), CD68 (clone KP1, Dako) after pre-treatment of the sections in a thermostatic bath at 96° C for 40 min in a pH 6 citrate buffer, CD5 (clone 4C7, Menarini) after pre-treatment of the sections in a thermostatic bath at 96° C for 40 min in a pH 8 EDTA buffer, S100 (polyclonal, Menarini), Calponin (polyclonal, UCS), pan-cytokeratin (MNF116, Dako), keratin 8 (35βH11, Dako), collagen IV (CIV94, Zymed, Histoline, Milan, Italy) after enzymatic pre-treatment. Diaminobenzidine (Menarini) was used as chromogenic substrate.

### Pathology description

**Figure 3:**
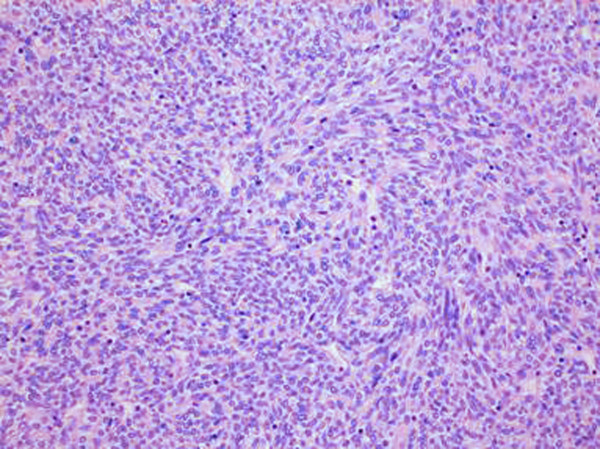
**Histopathology**. Histopathology showing a uniform growth of short, spindle shaped, mitotically inactive cells. Few small, typical lymphocytes were observed scattered or in small groups (Hematoxylin-Eosin stain, 20× magnification)

Under microscope, the tumour showed a uniform growth of short, spindle shaped, mitotically inactive cells. A few small, typical lymphocytes were seen scattered or in small groups (fig. 3).

An hemangiopericytic pattern was formed by the short, spindle-shaped cells with bland vesicular fused nuclei. In some areas a gap was found between the epithelial sheets and the tiny-walled vessels.

**Figure 4:**
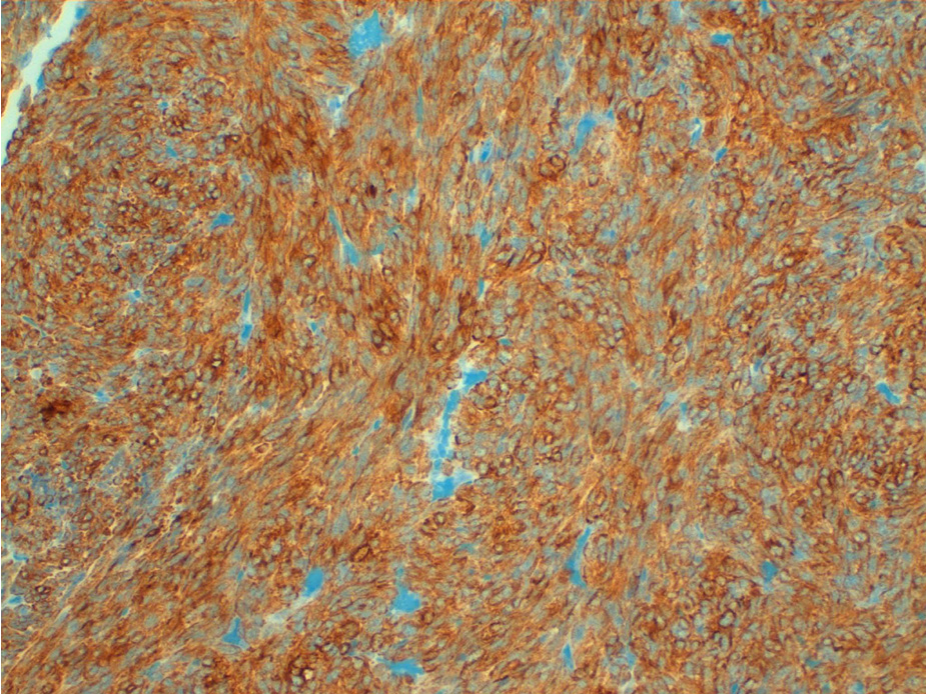
**Immunohistology for Cytokeratin**. Immunohistology showing positivity for Cytokeratin cocktails MNF116 20× magnification.

The immunohistochemical investigations tested negative for neuroectodermal (CD99, S100, HMB45), neuroendocrine (Chromogranin, Synaptophysin), vascular (CD31, CD34) and muscular (Calponin) markers, whereas the spindle cells were consistently positive for cytokeratin cocktails MNF116, AE1/AE3, for cytokeratin 19 and cytokeratin 7 (fig. 4).

**Figure 5:**
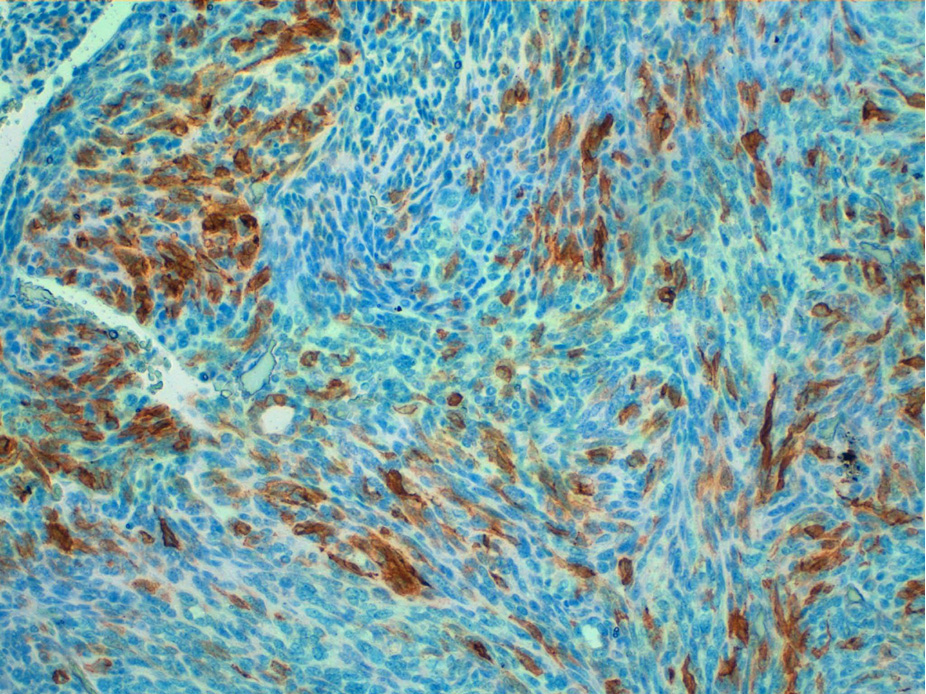
**Immunohistology for CD20**. Immunohistology showing positivity for CD20 20X magnification

**Figure 6:**
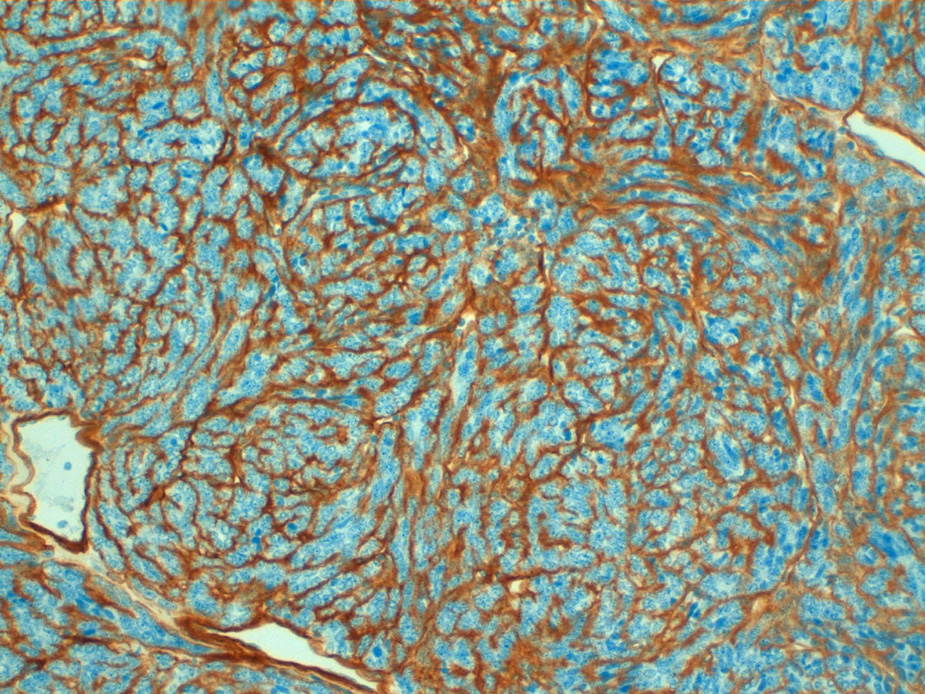
**Immunohistology for collagen IV**. Immunohistology showing positivity with a strong intercellular deposition of collagen IV 20× magnification

Furthermore, vimentin, epithelial membrane antigen (EMA), CD56, cytokeratin 8, BCL2 (week), were focally positive. The staining for CD20 showed an extensive multi-focal positive membranous staining of the spindle epithelial cells (fig. 5). The small lymphocytes were mature T lymphocytes CD3+, CD5+, negative for the immature T- lymphocyte markers CD1a, CD10, CD99. The staining for collagen IV showed strong intercellular deposition of the collagen among ribbons and nests of spindle cells (fig. 6). Varying from the tumour cells, scattered accessory cells were found to be positive for smooth muscle actin, S100, CD1a and CD68.

### Cytogenetic analysis

A search of the SYT translocation t(X;18)(p11;q11) (WHO 2002) as typical for synovial sarcoma, performed by fluorescent *In situ *hybridization (FISH) with the vysis probe of inter-phase nuclei in formalin-fixed, paraffin embedded sections showing negative results.

## Conclusions

In the posterior mediastinum, a variety of tumours occur, however the majority of them are of neurogenic origin, schwannomas and neurofibromas being the most frequently encountered neoplasms [6]. In our case, the para-vertebral site and the absence of clinical evidence for a metastatic spread suggested a localization of a primary tumour. Several entities were considered in the differential diagnosis, while no definite clinical, morphological and immunohistochemical character of diagnostic relevance was found for the entities considered. The possibility of a neurogenic origin was excluded because of the absence of a rich angiographic pattern and the neuroectodermal marker considered (S100).

The diagnosis of hemangiopericytoma was excluded because there were no typical spider-like radial branching vessels nor collagen IV and CD99 positivity.

A melanoma metastasis was excluded due to the bland cytological appearance, the lack of mitotic activity and the S100 and HMB45 negativity. Similarly, the possibility of a solitary fibrous tumour was ruled out on the basis of the CD34 negativity and of the week positivity [7] of bcl2.

The hypothesis that the lesion might have been a renal carcinoma metastasis, which appeared after the first surgery, was highly unlikely both from an angiographical aspect and the metabolic activity showed by FDG-PET. The diagnosis of Chordoma was excluded basing on FDG-PET activity as well as plasmocitoma that should have had no hyperfissation period in bone scan and high activity in FDG-PET.

In this case, the possibility of a synovial sarcoma was ruled out not only by the bland appearance of the nuclei, but by the lack of mitotic activity and the absence of the SYT translocation typically associated to Synovial sarcoma, as demonstrated by the FISH investigation.

In our case, the widespread positivity for Cytokeratins is in keeping only with epithelial tumours. Thymic epithelial tumours occur in several ectopic sites, such as inside or outside (particularly in cervical location) the thoracic cavity, according to the embryological derivation of the thymus from the IV branchial cleft. In the posterior mediastinum, the occurrence of ectopic thymoma has rarely been reported, but nevertheless the para-vertebral site has to be considered as a possible site for ectopic thymomas emerging. Nevertheless, in our case the lesion is clearly located in vertebral body and not in the posterior mediastinum, furthermore the bland appearance of the fusiform nuclei, the presence of scattered typical lymphocytes, the presence of peri-vascular spaces as typical for thymic epithelial tumours (TET), the strong collagen IV deposits among the spindle cells and the extensive co-expression of cytokeratins and CD20 were considered highly diagnostic for type A thymoma according to the World Class Organization classification system of TET [8]. The hemangiopericytic pattern is a frequent feature in spindle cell (type A) thymoma. Furthermore, the diagnostic value of the co-expression of cytokeratins and CD20 was considered. CD20 (L26), i.e. a fundamental marker of B-lymphocytes, was described as a frequent and specific marker of the medullary type of thymoma [9]. More recently, the frequency of the CD20 staining in spindle (medullary, WHO type A) and mixed spindle/lymphocytic (WHO type AB) thymoma has also been described as a specific feature of such thymoma types [10,11]. The presence of immature T lymphocytes is usually considered a feature related to the thymic origin of an epithelial tumour both inside and outside the thorax. However, the spindle cell, medullary (type A) thymomas are devoid of thymopoietic activity and the associated lymphoid component reflects the mature appearance and the mature T-cell phenotype belonging to the thymic medulla [12]. Type A and AB thymoma are usually characterized by a low grade of malignancy.

Established the thymic tissue origin of the lesion the metastatic nature was the most probable nevertheless it was excluded and the tumour considered primary because both FDG-PET and total body CT-SCAN didn't detect other lesions or ectopic thymus. The hypothesis is today confirmed by the checks performed at two years of follow-up.

The long history of the present case reflects this general tendency, although a local invasive capacity has been well documented. Furthermore, the case described points considering thymic origins for spindle cell tumours with a hemangiopericytic pattern of unknown derivation.

Considering the low grade of malignancy, the treatment chosen by the patient was acceptable for her disease. However an en-block vertebral resection would have decreased the risk of recurrence. A further treatment as radiation therapy was temporary excluded; the possibility to perform a second surgery and then radiation therapy was considered preferable according to the patient. In order to guarantee a correct histopathological interpretation as well as treatment, diagnosis has to be done based on patient history and clinical imaging.

## Competing interests

The authors declare that they have no competing interests.

## Authors' contributions

FM, MM and PV carried out the morphological and immunoistochemical studies, and participated in the sequence alignment and drafted the manuscript. CZ and NS participated in the design of the study and performed the statistical analysis. All authors read and approved the final manuscript.

## Pre-publication history

The pre-publication history for this paper can be accessed here:

http://www.biomedcentral.com/1471-2407/10/322/prepub
